# Platelets induce a proinflammatory phenotype in monocytes via the CD147 pathway in rheumatoid arthritis

**DOI:** 10.1186/s13075-014-0478-0

**Published:** 2014-11-18

**Authors:** Meng-yao Rong, Cong-hua Wang, Zhen-biao Wu, Wen Zeng, Zhao-hui Zheng, Qing Han, Jun-feng Jia, Xue-yi Li, Ping Zhu

**Affiliations:** Department of Clinical Immunology, State Key Discipline of Cell Biology, Xijing Hospital, Fourth Military Medical University, 127 West Changle Road, Xi’an, 710032 Shaanxi Province PR China; Department of Neurology, Tangdu Hospital, Fourth Military Medical University, 569 Xinsi Road, Xi’an, 710038 Shaanxi Province PR China

## Abstract

**Introduction:**

Activated platelets exert a proinflammatory action that can be largely ascribed to their ability to interact with monocytes. However, the mechanisms that promote dynamic changes in monocyte subsets in rheumatoid arthritis (RA) have not been clearly identified. The aim of this study was to determine whether platelet activation and the consequent formation of monocyte-platelet aggregates (MPA) might induce a proinflammatory phenotype in circulating monocytes in RA.

**Methods:**

The surface phenotype of platelets and the frequencies of monocyte subpopulations in the peripheral blood of RA patients were determined using flow cytometry. Platelets were sorted and co-cultured with monocytes. In addition, monocyte activation was assessed by measuring the nuclear factor kappa B (NF-κB) pathway. The disease activity was evaluated using the 28-joint disease activity score.

**Results:**

Platelet activation, circulating intermediate monocytes (Mon2) and MPA formation were significantly elevated in RA, especially in those with active disease status. Furthermore, Mon2 monocytes showed higher CD147 expression and responded to direct cell contact with activated platelets with higher cytokine production and matrix metallopeptidase 9 (MMP-9) secretion, which increased the expression of CD147. After the addition of specific antibodies for CD147, those effects were abolished. Furthermore, the NF-κB-driven inflammatory pathway may be involved in this process.

**Conclusions:**

These findings indicate an important role of platelet activation and the consequent formation of MPA in the generation of the proinflammatory cytokine milieu and for the promotion and maintenance of the pathogenically relevant Mon2 monocyte compartment in RA, which is likely to play an important role in the pathogenesis of autoimmunity.

## Introduction

Rheumatoid arthritis (RA) is a chronic inflammatory disease that is characterized by intense immune activation within the synovial compartment of joints and a variety of systemic manifestations. Monocytes and macrophages are key players in RA pathogenesis that secrete proinflammatory cytokines, such as tumor necrosis factor alpha (TNF-α) and interleukin 6 (IL-6) [1,2]. In the past, monocytes were considered to be a homogeneous population. Currently, at least three human monocyte populations can be defined by the expression of CD14, which is a part of the lipopolysaccharide (LPS) receptor [3], and the FcγIII receptor CD16 [4]. The CD14^++^CD16^+^ (‘intermediate’) monocyte subset remains the most poorly characterized because CD16^+^ monocytes had been analyzed as a single population until they were shown to comprise two subsets, the CD14^++^CD16^+^ cells and the CD14^+^CD16^++^ cells, and emerging functional and genetic evidence has uncovered their distinct roles [5-7]. However, because CD14^++^CD16^+^ and CD14^+^CD16^++^ monocytes overlap when they are defined solely by the presence of CD14/16 expression, an unequivocal discrimination is necessary. Based on current opinion, three subsets of monocytes, CD14++CD16–CCR2+ (‘classical’ (Mon1)), CD14++CD16 + CCR2+ (‘intermediate’ (Mon2)) and CD14 + CD16++CCR2– (‘nonclassical’ (Mon3)), have been defined [5-9]. Studies of autoimmune disorders that have reported monocyte subset perturbation have mostly involved RA [10-13] and Crohn’s disease (CD) [14-16]. Although the expansion of CD16+ monocytes in RA has been clearly demonstrated in several studies [17,18], it remained unclear until Rossol *et al*. reported a link between the increased frequency of the CD14^bright^CD16^+^ monocyte subpopulation in RA [10].

Recent studies have uncovered a novel platelet contribution to disease pathophysiology in inflammatory arthritis [19]. In humans, increased platelet activation is evident in patients with RA compared with healthy control subjects. CD147, which is a platelet receptor that is upregulated on activated platelets, is a critical mediator of the positive regulators that are implicated in platelet activation, and its binding to platelets fosters platelet degranulation. CD147 is a 57-kDa glycosylated transmembrane immunoglobulin that is also called extracellular matrix metalloproteinase inducer (EMMPRIN). CD147 is capable of homotypic binding, which can lead to platelet degranulation upon platelet binding and stimulation of the nuclear factor kappa B (NF-κB) pathway in monocytes, which lead in turn to the production of matrix metalloproteinase (MMP) and cytokines, specifically MMP-9, TNF-α and IL-6 [20]. Although platelet CD147 has been shown to be functional *in vitro*, little is known about platelet CD147 expression in RA patients.

Monocyte-platelet aggregates (MPA) are heterotypic complexes that are detectable in the peripheral blood and form in response to platelet activation [21]. Accordingly, the circulating MPA level increases in patients who have a variety of autoimmune disorders [22]. However, the importance of monocyte-platelet interactions in human inflammatory pathophysiology and the precise mechanisms by which such interactions modulate monocytic phenotype and function remain unclear. The aim of this study was to investigate platelet activation, the dynamic changes in the monocyte subsets and the association of MPA in RA patients. Second, we aimed to determine the underlying mechanism by which monocyte-platelet interactions modulate the phenotype and function of monocytes.

## Methods

### Patients

We enrolled 30 patients with active RA who satisfied the American College of Rheumatology/European League Against Rheumatism (ACR/EULAR) RA classification criteria [23]. The mean age of these active RA patients was 44 years (range 20 to 72), and the mean disease duration was 8 years. Patients had active disease that was consistent with the criteria of activity: (1) ≥ six joints were swollen; (2) ≥ six joints were tender; and (3) two of the following three criteria were satisfied: (a) on the day of the visit, morning stiffness lasted more than 45 minutes; (b) erythrocyte sedimentation rate (ESR) ≥28 mm/h; and (c) C-reactive protein (CRP) ≥20 mg/dl. All of the patients in the study were either untreated or treated only with nonsteroidal anti-inflammatory drugs. For all RA patients, a composite 28-joint disease activity score (DAS-28) was calculated to evaluate the disease status [24]. A DAS > 3.6 indicates an active disease status, and a DAS < 2.6 was considered to reflect remission. Normal control peripheral blood samples were taken from 10 healthy human donor volunteers who had no significant age or sex differences compared with the RA patients. The study was approved by the Ethics Committee of the Fourth Military Medical University. All of the patients and controls provided their informed consent to participate in the study.

### Reagents

Preconjugated fluorescent antibodies were from BD Biosciences (San Jose, CA, USA) (CD14–phycoerythrin (PE), P-selectin (CD62P)–PE, immunoglobulin G (IgG_1_)–PE, CD16–PE-Cy5, CD40L–PE, CD42a–fluorescein isothiocyanate (FITC), PAC-1–FITC, CD147–FITC, TNF-α–FITC, IL-6–FITC, CD61–PerCP, CCR2–Alexa Fluor and BrdU Flow Kit). Anti-CD147 antibody was provided by the Department of Cell Biology, Fourth Military Medical University (Xi’an, China). An anti-IKK beta (IKKβ) antibody was also used (Abcam, Cambridge, UK).

### Platelet isolation

Blood was diluted by the addition of Tyrode’s buffer at pH 6.5 (134 mM NaCl, 2.9 mM KCl, 0.34 mM Na_2_HPO_4_, 12 mM NaHCO_3_, 20 mM HEPES, 1 mM MgCl_2_, 5 mM glucose and 0.5 mg/ml bovine serum albumin (BSA)) and centrifuged at 600 × g for 3 minutes. Platelet-rich plasma (PRP) was further centrifuged for 2 minutes at 400 × g to pellet the contaminating red blood cells (RBCs). This solution was thereafter centrifuged for 5 minutes at 1300 × g to pellet the platelets. The platelets were resuspended in Tyrode’s buffer at pH 7.4 and quantified cytofluorometrically using anti-CD61 staining.

### Cell isolation and culture

The fasting blood samples were collected in acid citrate dextrose (ACD) anticoagulant tubes, and peripheral blood mononuclear cells (PBMCs) were prepared using a Ficoll density gradient for flow cytometric analysis [25]. Monocytes were prepared using Ficoll density gradient centrifugation and further isolated by positive selection using antibody-coupled microbeads (Miltenyi Biotec, Bergisch Gladbach, Germany) according to the manufacturer’s protocol. The purity of the monocyte preparation was analyzed using flow cytometry based on cell forward (FSC) and side scatter (SSC), and these cells amounted to 96% of the sample. The monocytes were suspended at a density of 10^6^ cells/ml in complete culture medium (RPMI 1640 supplemented with 100 U/ml penicillin and 100 μg/ml streptomycin, 2 mM glutamine and 10% heat-inactivated fetal calf serum; Gibco BRL/Life Technologies, Grand Island, NY, USA). Aliquots of purified platelets were added to the monocyte suspensions (1 ml final volume, monocyte: platelet ratio 1:100 in experiments where platelets were added) and incubated at 37°C in 5% CO_2_ for 48 hours.

### Flow cytometry

The procedure for staining the cell surface proteins was previously described [26]. Monocytes were first gated according to their FSC and SSC dot plots, and then three-color fluorescence was measured within the monocyte gate. Monocytes were subclassified according to CD14 and CD16 expression. The CD14^+^CD16^−^ (Mon1) cells were defined as monocytes expressing CD14 but not CD16. CD14^+^CD16^+^ monocytes were then further subclassified regarding CCR2 expression into CD14^++^CD16^+^CCR2^+^ (Mon2) and CD14^+^CD16^++^CCR2^−^ (Mon3). At least 20,000 events in the monocyte gate were measured per experiment. The results are presented as the percentages of positive cells. Circulating MPA was defined by double positivity for CD14 and CD42a. Intracellular staining of cytokines was performed using a BD Cytofix/Cytoperm Fixation and Permeabilization kit (BD Biosciences). Appropriately conjugated IgG antibodies were used as isotype controls. After washing with phosphate-buffered saline (PBS), the stained cells were subjected to flow cytometry analysis using a fluorescence-activated cell sorter (FACS) Calibur system and Cell Quest software (BD Biosciences).

### Monocyte cell function assays

Monocytes (1 × 10^6^ cells/ml) from normal donors were incubated with LPS, anti-CD147 monoclonal antibody [27] or IKK inhibitor (Cell Signaling Technology, Danvers, MA, USA), respectively, for 2 hours before co-culture or indirect co-culture. Monocyte co-culture with purified platelets (monocyte:platelet ratio 1:100 in experiments in which platelets were added) was incubated at 37°C, 5% CO_2_ for 48 hours. The indirect co-culture utilized Transwell culture chambers (Merck Millipore, Billerica, MA, USA) containing polycarbonate filters (pore size, 0.4 μm), allowing both cells to exchange soluble products through a semipermeable membrane but preventing cellular contact. Briefly, the monocytes (1 × 10^6^ cells/ml) were added to the upper chamber, while platelets (1 × 10^8^ cells/ml) or medium alone was added to the lower compartments. The proliferation of the monocyte subsets was measured using BrdU Flow Kits according to the manufacturer’s instructions.

### Gelatin zymography

Serum-free conditioned medium samples were mixed with sodium dodecyl sulfate (SDS) sample buffer and loaded onto a 10% polyacrylamide gel containing 0.1% gelatin. After electrophoresis, the gels were washed in 2.5% Triton X-100 for 30 minutes and incubated for 16 hours in reaction buffer. The gels were subsequently stained with 0.5% Coomassie Blue (R-250) and destained to visualize the zones of digestion as light areas against the darkly stained background. The gels were then scanned and analyzed using GeneSnap from SynGene Tools (Cambridge, UK).

### Statistical analysis

The presented results were representative of a minimum of three experiments and were expressed as the means ± standard deviations (SDs). Statistical analysis was conducted using Student’s *t* test or the Mann-Whitney *U* test as appropriate, and multiple comparisons with a single control were performed using analysis of variance (ANOVA) with Dunnett’s *t* test modification. GraphPad software (Cricket Software, Philadelphia, PA, USA) was used for the above analysis, and *P* values less than 0.05 were considered significant.

## Results

### High expression of CD147, PAC-1, CD62P and CD40L on platelets from RA patients

**Figure 1:**
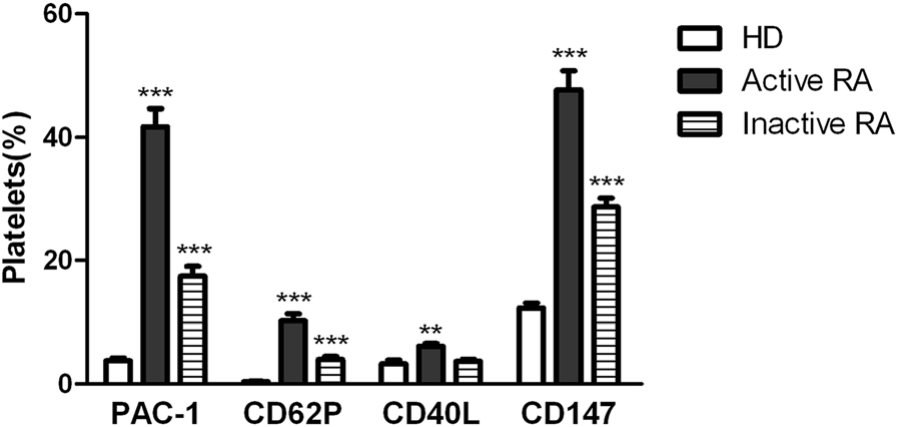
**High expression of CD147, PAC-1, CD62P and CD40L on platelets from RA patients.** PAC-1, CD62P, CD40L and CD147 expression as percentages of positive platelets in healthy controls (n = 10) and in patients with inactive RA (n = 10) and active RA (n = 30). (Means ± SDs of the means, ^**^ *P* <0.001 and ^***^ *P* <0.0001 vs. corresponding controls). RA: rheumatoid arthritis; SDs: standard deviations.

**Figure 2:**
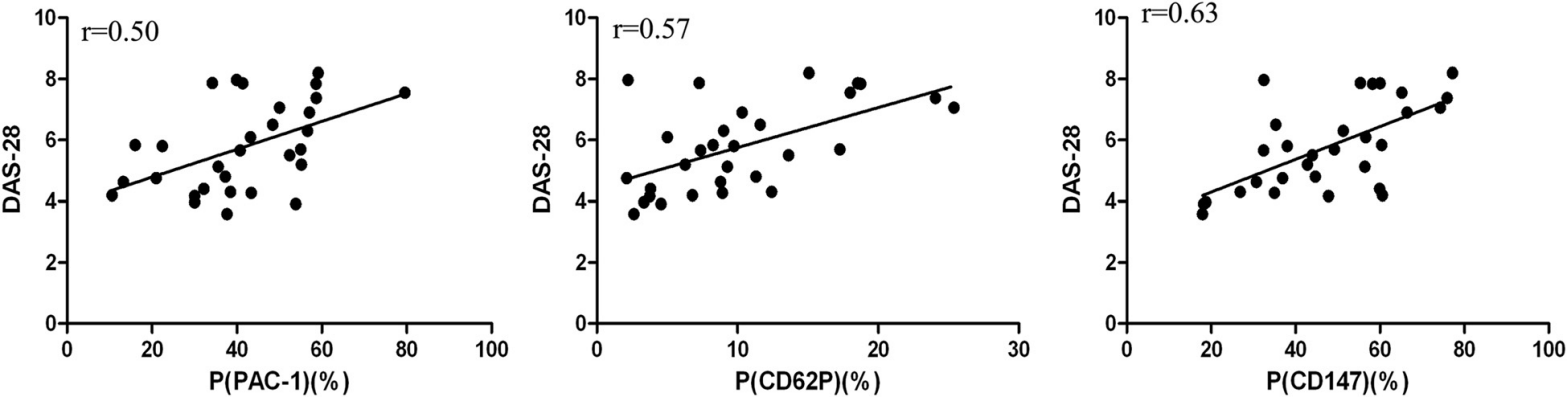
**DAS-28 was positively correlated with the expression levels of PAC-1, CD62P and CD147 within active RA patients.** DAS-28 vs. PAC-1 (r = 0.50, P = 0.0048), vs. CD62P (r = 0.57, P = 0.0009) and vs. CD147 (r = 0.63; P = 0.0002) expression as a percentage of positive platelets. DAS-28: composite 28-joint disease activity score; P: platelet; RA: rheumatoid arthritis.

Flow cytometry showed the percentage of cells that stained positive for CD147, PAC-1 and P-selectin (CD62P); the percentages of stained active (mean ± SD 47.63 ± 3.1, 41.73 ± 2.89 and 10.18 ± 1.15%, respectively) platelets and platelets from patients with inactive RA (28.69 ± 1.42, 17.41 ± 1.63 and 3.99 ± 0.46%, respectively) were significantly higher than those of healthy platelets (12.26 ± 0.83, 3.8 ± 0.44 and 0.39 ± 0.07%, respectively; *P* <0.05), especially in active RA patients. The percentage of active RA platelets that stained positive for CD40L (6.11 ± 0.44%) was higher than that of healthy platelets (3.3 ± 0.56%; *P* <0.05), but the value for patients with inactive RA was not significantly different from that of healthy platelets (3.66 ± 0.36%; *P* >0.05) (Figure 1). Flow cytometry showed the percentages of cells that stained positive for CD147, PAC-1, CD62P and CD40L in active RA platelets, which were higher than those of inactive RA platelets (*P* <0.01). Furthermore, the percentage of platelets that were positive for CD147 expression was considerably higher than those that were positive for PAC-1, CD62P and CD40L expression in the overall population that was examined (Figure 1). Disease activity, which was evaluated using DAS-28, was also significantly and positively correlated with the expression of PAC-1, CD62P and CD147 (r = 0.50, r = 0.57 and r = 0.63, respectively; *P* <0.05) within the group of patients with active RA (Figure 2).

### High percentages of Mon2 and Mon3 and the expression of CD147 in peripheral blood from RA patients

**Figure 3:**
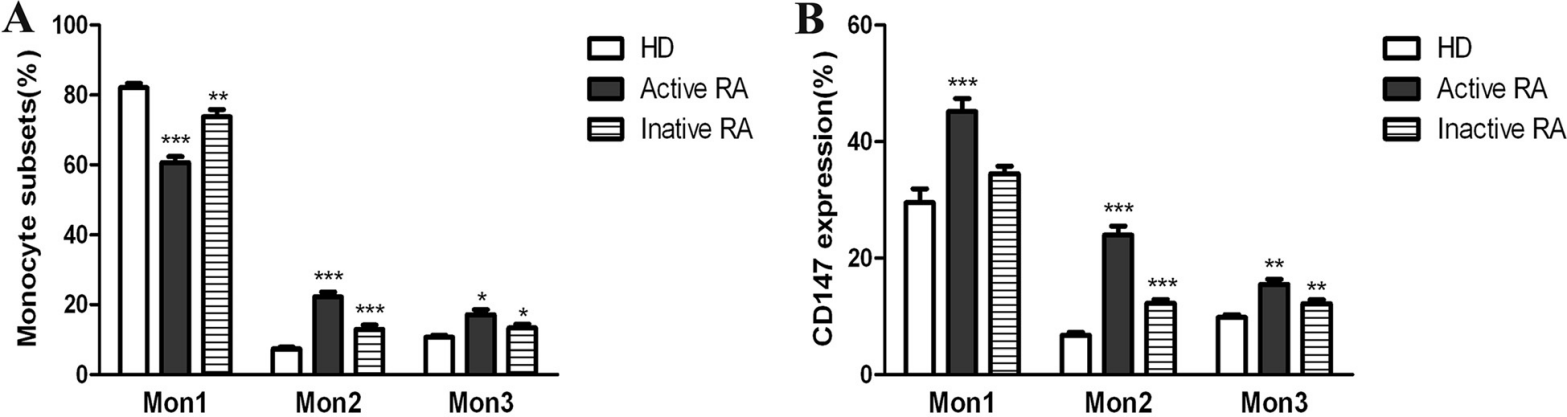
**High expression of Mon2, Mon3 and CD147 in the peripheral blood of RA patients. (A)** The percentages of the monocyte subsets and **(B)** the expression of CD147 in healthy controls (n = 10) and patients with inactive RA (n = 10) and patients with active RA (n = 30). (Means ± SDs of the mean,^*^ *P* <0.05, ^**^ *P* <0.001 and ^***^ *P* <0.0001 vs. corresponding controls). Mon1: CD14^++^CD16^−^CCR2^+^; Mon2: CD14^++^CD16^+^CCR2^+^; Mon3: CD14^+^CD16^++^CCR2^−^; RA: rheumatoid arthritis; SDs: standard deviations.

Monocytes were subclassified into CD14^++^CD16^−^CCR2^+^ (Mon1), CD14^++^CD16^+^CCR2^+^ (Mon2) and CD14^+^CD16^++^CCR2^−^ (Mon3). The frequencies of Mon2 and Mon3 monocytes in peripheral blood samples from patients with active RA (22.2 ± 1.45 and 17.17 ± 1.41%, respectively) or patients with inactive RA (12.87 ± 1.27 and 13.33 ± 1.04%, respectively) were higher than the percentages in the healthy control group (7.26 ± 0.51 and 10.64 ± 0.56%, respectively, *P* <0.05), especially for Mon2 (Figure 3A). A detailed analysis of the demographic and clinical parameters in the RA patients revealed no statistically significant correlations for the expansion of Mon2 monocytes (data not shown).

The expression of CD147 was increased in all three subsets of monocytes from patients with RA, compared with those of the healthy control subjects (Figure 3B). The proportion of CD147 expression in the Mon1, Mon2 and Mon3 monocytes from patients with active RA (45.15 ± 2.28, 24 ± 1.52 and 15.46 ± 0.911%, respectively) or patients with inactive RA (34.47 ± 1.33, 12.24 ± 0.61, 12.19 ± 0.65%, respectively) was significantly increased for all three subsets compared with those of the healthy controls (29.47 ± 2.39, 6.74 ± 0.51 and 9.79 ± 0.46%, respectively), except for the proportion of CD147 expression on Mon1 monocytes in patients with inactive RA. The proportion of CD147 expression on Mon2 monocytes in active or patients with inactive RA compared with that of the healthy controls was significantly (3.56-fold or 1.82-fold, respectively) higher than the proportion of Mon1 (1.55-fold or 1.17-fold, respectively) and Mon3 (1.58-fold or 1.25-fold, respectively) cells (Figure 3B).

### High MPA levels and positive correlations with Mon2 and with the expression of CD147 on platelets and Mon2

**Figure 4:**
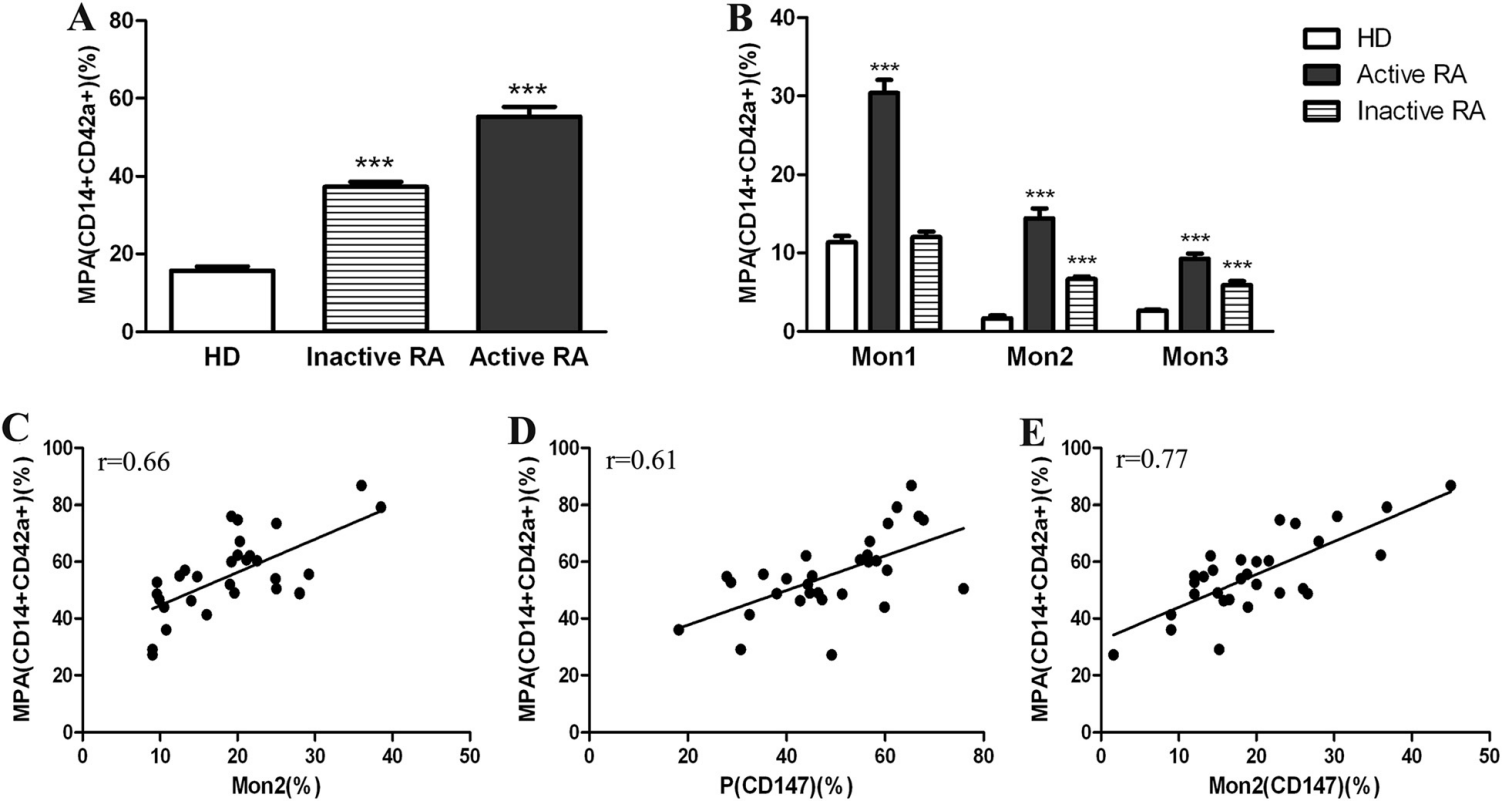
**High MPA levels and positive correlations with Mon2 and with the expression of CD147 on platelets and Mon2. (A)** The percentages of platelets that aggregated with monocytes and **(B)** the monocyte subsets in healthy controls (n = 10), patients with inactive RA (n = 10) and patients with active RA (n = 30). MPA levels **(C)** vs. the percentage of Mon2 (r = 0.66, P <0.0001), **(D)** vs. the expression of CD147 on platelets (r = 0.61, P = 0.0004) and **(E)** vs. the expression of CD147 on Mon2 monocytes (r = 0.77, P <0.0001) within the group of patients with active RA (means ± SDs of the mean, ^*^ *P* <0.05, ^**^ *P* <0.001 and ^***^ *P* <0.0001 vs. corresponding controls). HD: healthy donors; P: platelet; MPA: monocyte-platelet aggregates; Mon1: CD14^++^CD16^−^CCR2^+^; Mon2: CD14^++^CD16^+^CCR2^+^; Mon3: CD14^+^CD16^++^CCR2^−^; RA: rheumatoid arthritis.

Patients with RA (active or inactive), compared with the healthy controls, had significantly higher numbers of circulating MPA (55.36 ± 2.53 and 37.32 ± 1.22, respectively vs. 15.69 ± 3.33, mean ± SD; *P* <0.0001) (Figure 4A). Moreover, the proportions of Mon1, Mon2 and Mon3 monocytes that aggregated with platelets in patients with active RA (30.38 ± 1.65, 14.41 ± 1.28 and 9.23 ± 0.68%, respectively) or patients with inactive RA (12 ± 0.72, 6.65 ± 0.33 and 5.87 ± 0.53%, respectively) compared with the healthy controls (11.34 ± 0.82, 1.68 ± 0.39 and 2.67 ± 0.16%, respectively) were all significantly increased (*P* <0.0001), except for the proportion of Mon1 monocytes that aggregated with platelets in patients with inactive RA (Figure 4B). The proportion of Mon2 monocytes that aggregated with platelets in patients with active or inactive RA compared with healthy controls was significantly (8.6-fold or 4-fold, respectively) higher than the proportion of aggregated Mon1 (2.7-fold or 1.1-fold, respectively) and Mon3 monocytes (3.5-fold or 2.2-fold, respectively).

When examining the relationships between MPA levels and other variables (the percentage of Mon2, the expression of CD147 on platelets and the expression of CD147 on Mon2), a linear correlation was found between the MPA levels and the percentage of Mon2 monocytes (r = 0.66, P <0.0001) (Figure 4C), the expression of CD147 on platelets (r = 0.61, P = 0.0004) (Figure 4D) and the expression of CD147 on Mon2 monocytes (r = 0.77, P <0.0001) (Figure 4E). However, the levels of MPA correlated more closely with the percentage of expressed CD147 on Mon2 monocytes than the percentage of Mon2 monocytes that were present in individual patients, whereas no such correlation was observed in healthy control subjects. The levels of MPA did not correlate with the percentages of Mon1 or Mon3 monocytes (data not shown).

### Platelet-monocyte interactions

**Figure 5:**
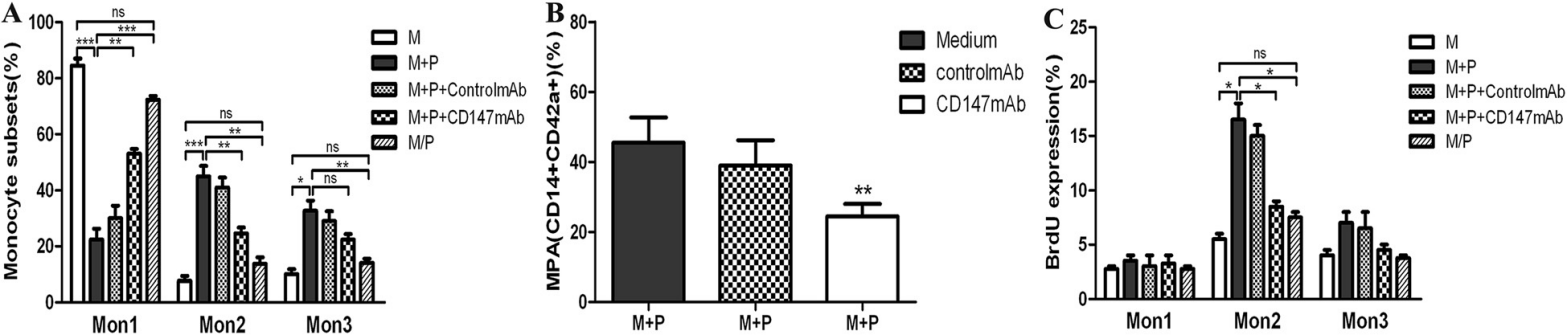
**Platelet-monocyte interactions.** Changes in the percentage of monocyte subsets **(A)**, MPA **(B)** and intracellular expression of BrdU **(C)** in monocytes contact co-cultured (M + P) or no cellular contact co-cultured with platelets from RA patients (M/P), expressed as percentages of monocyte subsets and CD14^+^CD42a^+^ (MPA) cells, respectively, compared to monocytes cultured in medium alone (M) after 48 h. Also shown are the effects of anti-CD147 blocking antibody (CD147mAb) or nonblocking control antibody (control mAb) added to monocyte-platelet co-culture (n = 4 for each). (^*^ *P* <0.05, ^**^ *P* <0.001 and ^***^ *P* <0.0001 vs. corresponding controls). MPA: monocyte-platelet aggregates; P: platelet; RA: rheumatoid arthritis.

To directly test whether intercellular contact is required for differentiation of the monocyte subpopulations, monocytes (1 × 10^6^ cells/ml) from normal donors were incubated with or indirectly co-cultured with RA platelets (1 × 10^8^ cells/ml). The indirect co-culture utilized Transwell culture chambers, which allows both cells to exchange soluble products through a semipermeable membrane but prevents cellular contact. We found that the percentage of Mon2 was higher when co-cultured with platelets from the RA patient group (45 ± 3.6%, *P* <0.001) compared with the no cellular contact (13.67 ± 2.33%, *P* <0.01) (Figure 5A), CD147-blocking monoclonal antibody (mAb) (24.67 ± 2.03%, *P* <0.01) or control groups (7.63 ± 1.71%). The percentage of Mon3 was also higher in the co-culture with platelets from the RA patient group (32.67 ± 3.71%, *P* <0.01) compared with the no cellular contact (14.00 ± 1.53%, *P* <0.01) and control groups (10.03 ± 1.78%), but a significant (5.9-fold) increase in Mon2 was observed (Figure 5A). Blockade of CD147 abrogated MPA formation from monocytes in the presence of platelets (Figure 5B) and also markedly reduced the percentage of Mon2 (Figure 5A). Following co-culturing with platelets, the intracellular BrdU levels were significantly increased in the Mon2 monocyte subsets (16.5 ± 1.5%, *P* <0.05) compared with the control (5.5 ± 0.5%), CD147-blocking mAb (8.5 ± 0.5%) or no cellular contact groups (7.5 ± 0.5%) (Figure 5C).

### CD147 on platelets stimulates cytokine expression and MMP-9 production of monocytes via NF-κB activation

**Figure 6:**
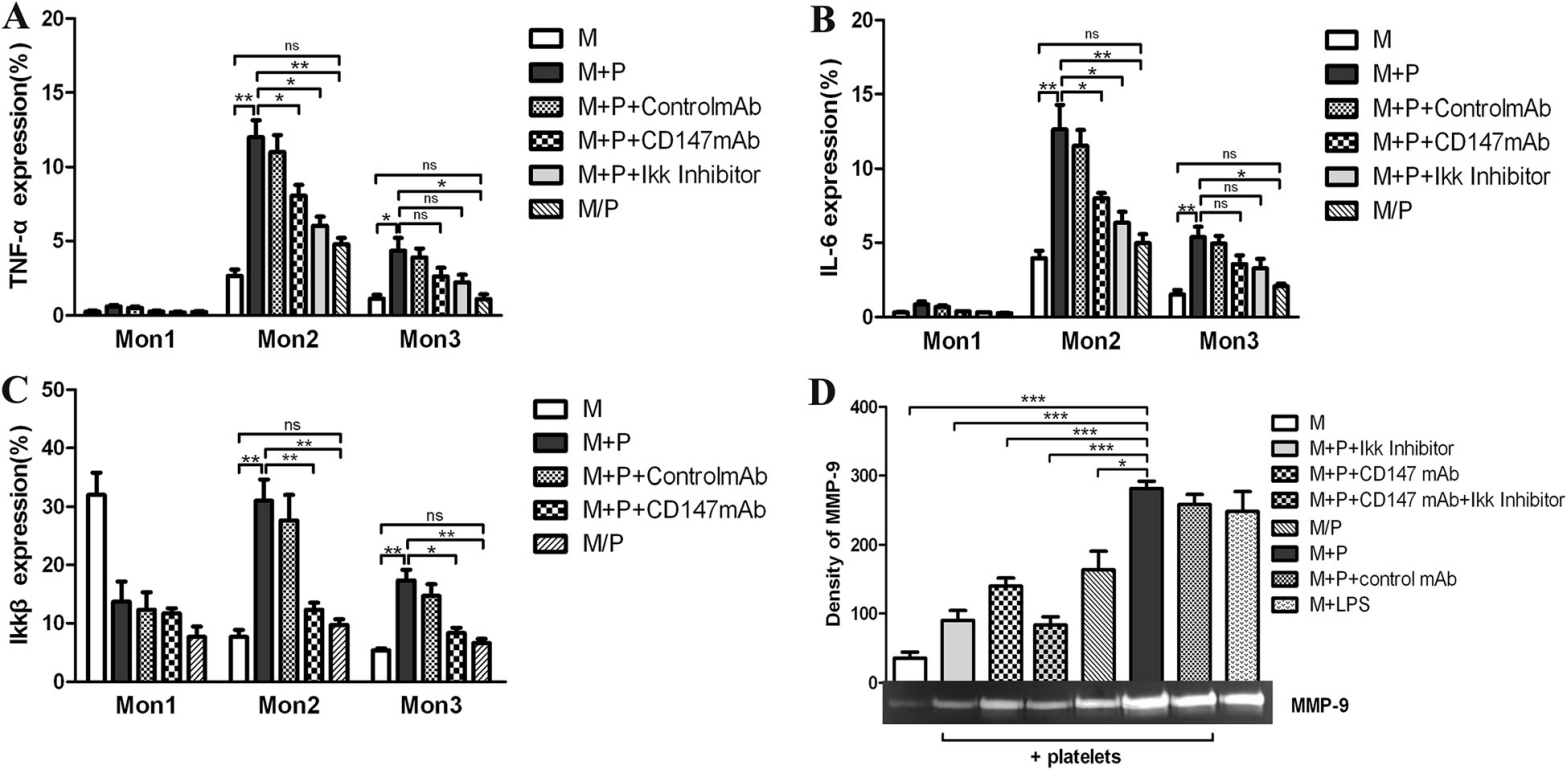
**CD147 on platelets stimulates cytokine expression and MMP-9 production of monocytes by NF-κB activation.** Isolated human monocytes were pretreated with medium (M), with anti-CD147-blocking antibody (CD147mAb), with IKK inhibitor, with nonblocking control antibody (control mAb) and with lipopolysaccharide (LPS, positive control) for 24 hours. Thereafter, monocytes were washed and contact co-cultured (M + P) or no cellular contact co-cultured (M/P) with freshly isolated platelets from RA patients for an additional 24 hours. Subsequently, intracellular expression of TNF-α **(A)**, IL-6 **(B)** and IKKβ **(C)** in monocyte subsets and MMP-9 activity **(D)** were determined (mean ± standard error of the mean, *P* >0.05, ^*^ *P* <0.05, ^**^ *P* <0.001 and ^***^ *P* <0.0001 vs. corresponding controls). IL-6: interleukin 6; LPS: lipopolysaccharide; mAb: monoclonal antibody; MMP: matrix metalloproteinases; MPA: monocyte-platelet aggregates; NF-κB: nuclear factor kappa B; RA: rheumatoid arthritis; TNF-α: tumor necrosis factor alpha.

In this study, the intracellular TNF-α and IL-6 levels in Mon2 monocytes were higher compared with the levels in Mon1 and Mon3 monocytes. Following co-culturing with RA platelets, the highest production levels of TNF-α and IL-6 were detected in Mon2 monocytes (12 ± 1.16% and 12.63 ± 1.68%, respectively; *P* <0.01), whereas their expression levels were significantly lower in the no cellular contact (4.77 ± 0.43% and 4.97 ± 0.61%, respectively; *P* <0.01), anti-CD147-blocking antibody (8.07 ± 0.72% and 8.00 ± 0.36%, respectively; *P* <0.05), IKK inhibitor (6.03 ± 0.61% and 6.33 ± 0.75%, respectively; *P* <0.05) and control groups (2.83 ± 0.6% and 4.07 ± 0.58%, respectively; *P* <0.01) (Figure 6A, B). Following co-culturing with platelets, the intracellular IKKβ levels were significantly increased in the Mon2 and Mon3 monocyte subsets (31 ± 3.61% and 17.33 ± 1.86%, respectively) compared with the control (7.67 ± 1.2% and 5.33 ± 0.33%, respectively), anti-CD147-blocking antibody (12.33 ± 1.2% and 8.3 ± 0.88%, respectively) or no cellular contact groups (9.73 ± 0.93% and 6.6 ± 0.7%, respectively) (*P* = 0.0036, *P* = 0.008 and *P* = 0.0047, respectively, for Mon2; *P* = 0.0031, *P* = 0.0119 and *P* = 0.0057, respectively, for Mon3) (Figure 6C).

To investigate the capacity of CD147 on the platelet surface to activate proteases, we co-incubated isolated human platelets with isolated monocytes in which the surface expression of CD147 had been reduced by pretreatment with CD147-blocking mAb. We found that the interplay between freshly isolated RA platelets and monocytes amplified MMP-9 activity (Figure 6D). In fact, the CD147-blocking mAb-induced inhibition of CD147 expression on monocytes reduced this effect, suggesting that platelet-monocyte interactions contribute to MMP-9 secretion. To test the hypothesis that platelet-monocyte interactions evoke protease stimulation by activating NF-κB, we pretreated human isolated monocytes with an IKK inhibitor and then incubated them with platelets. As depicted in Figure 6C, co-culturing with platelets led to the activation of MMP-9, which was hindered by inhibition of the IKK complex. Stimulation with bacterial LPS was used as a positive control. Together, these results indicate that CD147 stimulates MMPs and activates broader inflammatory processes via NF-κB translocation with the prevailing proinflammatory responses.

## Discussion

RA is a chronic, autoimmune, inflammatory disease that primarily affects synovial joints, and its cause remains obscure. The aim of this study was to determine if platelets induced the proinflammatory phenotype of monocytes in RA. Elevated CD14^high^CD16^+^ monocyte frequencies were found in patients with active RA [10]. However, because the CD14^++^CD16^+^ and CD14^+^CD16^++^ subsets overlap when they are defined solely by the presence of CD14/16 expression, an unequivocal discrimination is necessary. In keeping with recent data and statements of the consensus on the nomenclature of monocyte subsets, we observed that two phenotypically and functionally distinct CD16^+^ subsets can be reliably discriminated based upon their expression of CCR2. CD14^++^CD16^+^CCR2^+^ (Mon2) cells are a distinct population of monocytes that can be distinguished from the classic CD14^++^CD16^−^CCR2^+^ (Mon1) and nonclassical CD14^+^CD16^++^CCR2^−^ (Mon3) monocyte populations. Our study showed high frequencies of Mon2 monocytes in peripheral blood samples from active RA patients, which was consistent with the results of other studies that linked Mon2 monocytes to other inflammatory diseases [28], and suggests a proinflammatory role for these cells. Moreover, the increased frequency of Mon2 monocytes might be an indirect consequence of fewer circulating Mon1 monocytes, which are considered less mature than Mon2 monocytes, indicating that circulating monocytes in RA are globally more mature than in healthy donors, or because Mon1 monocytes infiltrated the inflamed joints and thus are less represented in the circulation. However, the mechanisms of the high frequencies of Mon2 monocytes in RA patients are unknown and deserve further exploration. A previous study showed that CD147 is preferentially expressed on activated inflammatory cells, such as monocytes or foam cells. We hypothesized that the increased surface expression of CD147 on Mon2 monocytes is a further indication for their involvement in inflammatory immune responses.

The platelet expression of CD147 has been reported [21] and may have various biological functions, including the induction of MMP expression. We report the high expression of CD147 on circulating platelets in RA patients. We further show marked associations among platelet CD147 surface expression, monocyte (and their individual subsets) CD147 expression, inflammatory markers and traditional markers of platelet activation, such as CD62P and PAC-1. Furthermore, with the use of whole-blood flow cytometry to minimize artifactual activation due to handling, the percentage of platelets that were positive for CD147 was much greater than the percentage that was positive for CD62P or PAC-1, which suggests that circulating platelets may express CD147 in a more constitutive manner than their expression of CD62P or PAC-1. Platelet CD147 may be a unique marker of the effect of disease activity on platelet activation status. Moreover, we found a positive correlation between CD147 expression on circulating platelets and the DAS-28 score, which is in agreement with our finding of traditional markers of platelet activation in active RA patients. In addition, CD147 is capable of homotypic binding, which can lead to platelet degranulation upon platelet binding [21]. These findings suggest that CD147 expression on platelets may be of pathogenic relevance to RA. Our findings also indicate that the high expression of CD147 on platelets that correlated with disease activity parameters might reflect the degree of systemic inflammation and could be a potential biomarker for disease activity in RA patients.

The formation of MPA has been reported to be increased in patients with RA. In our study, a linear correlation was found between MPA levels and the expression of CD147 on platelets. In the present study, we demonstrated that the numbers of Mon2 monocytes increased during the active phase of RA in the presence of activated platelets and the consequent MPA formation, and that anti-CD147 blocking antibody group, which we found to abrogate MPA formation and phenotypic shifts, appears to be induced by contact co-culturing of platelets and monocytes. Although other groups have reported that platelets can alter monocytic CD16 expression [29], this observation has been interpreted as an effect on the terminal monocytic maturation toward macrophages. Our *in vitro* results, together with our co-culture model data, demonstrate that physical contact of monocytes with platelets is critical in this process, most likely by CD147/CD147 interactions between these cells, triggering a change in the circulating monocyte phenotype rather than a terminal differentiation event. Furthermore, our study found a linear correlation among MPA levels, the percentage of Mon2 monocytes and the expression of CD147 on Mon2, which suggests that platelets are functionally linked to the observed expansion of the Mon2 subpopulation in RA. BrdU experiments confirmed these results and verified that CD147-upregulated platelets may act as amplifiers of the pathogenetic cascade by directly contacting monocytes.

In this study, we found that the contact co-culturing of platelets and monocytes resulted in an increase in the percentage of Mon2 monocytes but no difference in the noncontact co-culture group or on the effect of Mon2 and Mon3 monocytes that was abrogated by CD147 mAb in the CD147-blocking mAb group. In addition, Mon2 monocytes have more elevated intracytoplasmic levels of proinflammatory cytokines (including IL-6 and TNF-α) than Mon1 and Mon3 monocytes, which is in keeping with their ability to produce a modified proinflammatory response. The correlation between platelets and monocytes may be explained by the concurrent activation of CD147 on the platelet and monocyte populations, especially Mon2 monocytes, by common agonists. For example, homotypic binding to monocyte CD147 induces the production of IL-6 and TNF-α [21]. Some of these factors are likely to cause platelet activation [30-32] and would be expected to correlate with RA disease activity, which we show here in accordance with platelet CD147 expression. Furthermore, Zhou *et al*. demonstrated that CD147 expression is upregulated during the differentiation of monocytes into macrophages and that this upregulation induces the secretion and activation of MMP-2 and MMP-9 and enhances the invasive ability of monocytes [33]. This finding is in agreement with our finding that monocyte-derived MMP-9 activity was induced more in contact co-cultures with RA platelets with a high expression of CD147. In accordance with Galt *et al*. [27], the CD147-blocking mAb reduced this effect, which suggested that CD147-CD147 binding contributes to MMP-9 secretion during platelet-monocyte interactions. Together, these results indicate that CD147 on the platelet surface interacts with CD147 on monocytes to stimulate MMP-9 activity and that CD147 may represent a novel target to diminish the burden of protease activity and inflammation in RA.

Although CD147 plays an important role in the interaction between Mon2 monocytes and platelets, the pathways by which CD147 mediates intracellular activation are not well understood. We showed that CD147 activates NF-κB, which, in turn, leads to the production of MMP-9 and proinflammatory cytokines (including IL-6 and TNF-α), which can be reduced by adding either CD147 mAb or NF-κB inhibitors *in vitro*. These data support our previous observation of the increased surface expression of CD147 on circulating monocytes in RA [34], which is a condition that is associated with increased plasma levels of IL-6 and TNF-α [35-37]. Moreover, although the NF-κB pathway was activated in the Mon2 and Mon3 monocyte subsets (that is, an increase in intracellular IKKβ levels) following contact co-culture of platelets and monocytes, the highest value of this key transcriptional factor was observed in Mon2, which was due to their prominent function in the promotion and generation of the proinflammatory cytokine milieu. To the best of our knowledge, a regulatory function of CD147 in monocyte subsets and the production of inflammatory cytokines have not been described for RA. Thus, these data support the concept that CD147-mediated cellular interaction between CD147 on the cell surface regulates broad, NF-κB-related Mon2 monocyte activities rather than pure MMP activity. Furthermore, CD147 may play an important role in systemic inflammation and thus represents a potential future therapeutic anti-inflammatory target.

## Conclusions

In summary, the findings of this study extend the role of CD147 expression on platelets in RA. Moreover, these findings indicate an important role of Mon2 monocytes in the promotion and generation of the proinflammatory cytokine milieu, which is strongly related to and may be driven by the extent of platelet activation. Our findings shed new light on the relevance of platelet-monocyte interactions in the pathophysiology of RA, and CD147 may play an important role in this process via the NF-κB pathway. The inhibition of CD147 may be a promising target for novel therapeutic strategies in RA patients.

## Abbreviations

ACD: acid citrate dextrose
BSA: bovine serum albumin
CRP: C-reactive protein
DAS-28: 28-joint disease activity score
EMMPRIN: extracellular matrix metalloproteinase inducer
ESR: erythrocyte sedimentation rate
FSC: forward scatter
IgG: immunoglobulin G
IL-6: interleukin 6
LPS: lipopolysaccharide
mAb: monoclonal antibody
Mon1: CD14^++^CD16^−^CCR2^+^ monocytes
Mon2: CD14^++^CD16^+^CCR2^+^ monocytes
Mon3: CD14^+^CD16^++^CCR2^−^ monocytes
MMPs: matrix metalloproteinases
MPA: monocyte-platelet aggregates
NF-κB: nuclear factor kappa B
PBMC: peripheral blood mononuclear cell
PBS: phosphate-buffered saline
PRP: platelet-rich plasma
SSC: side scatter
RA: rheumatoid arthritis
RBCs: red blood cells
SD: standard deviation
TNF-α: tumor necrosis factor alpha
